# Establishing entrustable professional activities for psychiatry residents in China

**DOI:** 10.1186/s12909-023-04583-9

**Published:** 2023-09-01

**Authors:** Feng Yan, Xu Yang, Ligang Zhang, Huaqin Cheng, Luyuan Bai, Fude Yang

**Affiliations:** 1 Beijing Huilongguan Hospital, Peking University Huilongguan Clinical Medical School, Beijing, China; 2 Institute of Medical Education &National center for Health Professions Education Development, PeKing University, Beijing, 100083 China

**Keywords:** Entrustable professional activities, Psychiatry, Standardized resident training, Medical education, Competency evaluation

## Abstract

**Purpose:**

The authors established entrustable professional activities for psychiatry residents in China.

**Methods:**

The authors conducted a literature research and two expert consultation rounds following the Delphi method in 2022 to screen and optimize entrustable professional activities for psychiatry residents.

**Results:**

The effective questionnaire recovery rate in the two consultation rounds was 100% (44/44). The expert authority coefficients of the first and second consultation rounds were 0.861 and 0.881, respectively. The Kendall harmony coefficients of the first and second expert consultation rounds were 0.279 (χ^2^ = 405.43, *P* < .001) and 0.389 (χ^2^ = 3456.83, *P* < .001), respectively. The arithmetic means of the various indicators’ evaluation results in the two consultation rounds ranged between 3.61 and 4.93, and the full score rates were between 13.6% and 93.2%. The authors established 17 entrustable professional activities for psychiatry residents and their contents with phase-based modularization and formulated the entrustable level of each at various stages.

**Conclusions:**

Combined with standardized psychiatry training characteristics, the authors preliminarily established phase-specific and modular entrustable professional activities for psychiatry residents. The formulated entrustable professional activities are suitable for the practice and clinical environment of standardized psychiatry training in China. The devised system has good observability and measurability and provides a simple and feasible competency evaluation method for standardized psychiatry resident training.

**Supplementary Information:**

The online version contains supplementary material available at 10.1186/s12909-023-04583-9.

## Introduction

In 2010, the Commission on the Education of Health Professionals for the 21st century proposed the third-generation reform of medical education, emphasizing the competency-based medical education (CBME) training mode [1]. Since 2014, China has implemented a nationwide standardized resident training system that is gradually maturing. Medical education professionals in China have started to realize the importance of cultivating the competence of clinicians. Some scholars used behavioral event interviews, expert consultations, and questionnaire surveys to build a competency model suitable for clinicians in China [2]. However, its implementation and evaluation were too cumbersome, resulting in weakened clinical diagnosis and treatment integrity. The trainees cannot effectively integrate various abilities into clinical work, and such models show difficulties in achieving the expected practical effects [3, 4].

In 2005, Professor ten Cate of the Medical Center of Utrecht University in the Netherlands first introduced the concept of entrustable professional activities (EPAs) [5]. EPAs emphasize medical behavior integrity and direct observations based on the observable, measurable, and executable clinical working environment [6]. They have been widely adopted internationally and are considered a good CBME evaluation method [7]. At present, EPAs are implemented in the United States, Australia, New Zealand, the Netherlands, Canada, and other countries and are included in several postgraduate medical education courses, including in pediatrics, internal medicine, family practice, psychiatry, obstetrics and gynecology, and nursing [8]. Papers reported mainly EPA development and/or EPA implementation studies whereas EPA assessment studies were less frequent [9], including three studies on constructing psychiatry EPAs [10–12], six on implementing EPAs [13–18], and one on constructing and implementing EPAs [19]. A relatively complete framework and implementation experience in psychiatry EPAs are available only in the United States, Australia, New Zealand, and Canada [18–20]. As a theoretical framework under exploration, many aspects of psychiatry EPAs are still worth investigating and improving. An EPAs evaluation system suitable for China’s national conditions has not been established. Based on the current standardized resident training situation at Huilongguan Clinical Medical College of Peking University, we explored establishing EPAs for competency evaluation of psychiatry residents on the basis of twelve tips to develop entrustable professional activities [21], providing some reference for daily and phase-based psychiatry competency evaluations during postgraduate medical education.

## Methods

### Research methods

We conducted a literature research and two expert letter consultation rounds following the Delphi method [22] to establish entrustable professional activities for psychiatry residents in China. The study was performed in accordance with the latest version of the Declaration of Helsinki and approved by the ethics committee of Beijing Huilongguan Hospital.

### Composition of the research group

The research group was composed of ten people, one with a senior professional title, four with a deputy senior professional title, and five with an intermediate professional title. One had a doctoral degree and nine had a master’s degree. Specifically, the team included the director of the education department, the ward director, and eight senior clinical front-line teachers, all with over ten years of teaching experience. The group members are all psychiatrists who have undergone two rounds of training in the early stages and are familiar with the EPA concept. The main team responsibilities included literature research, formulating the consultation form, expert selection, preparing an online Questionnaire Star version, distributing and recovering the consultation forms, index screening and revision, statistically analyzing the data, and writing this manuscript.

### Literature research

**Fig. 1:**
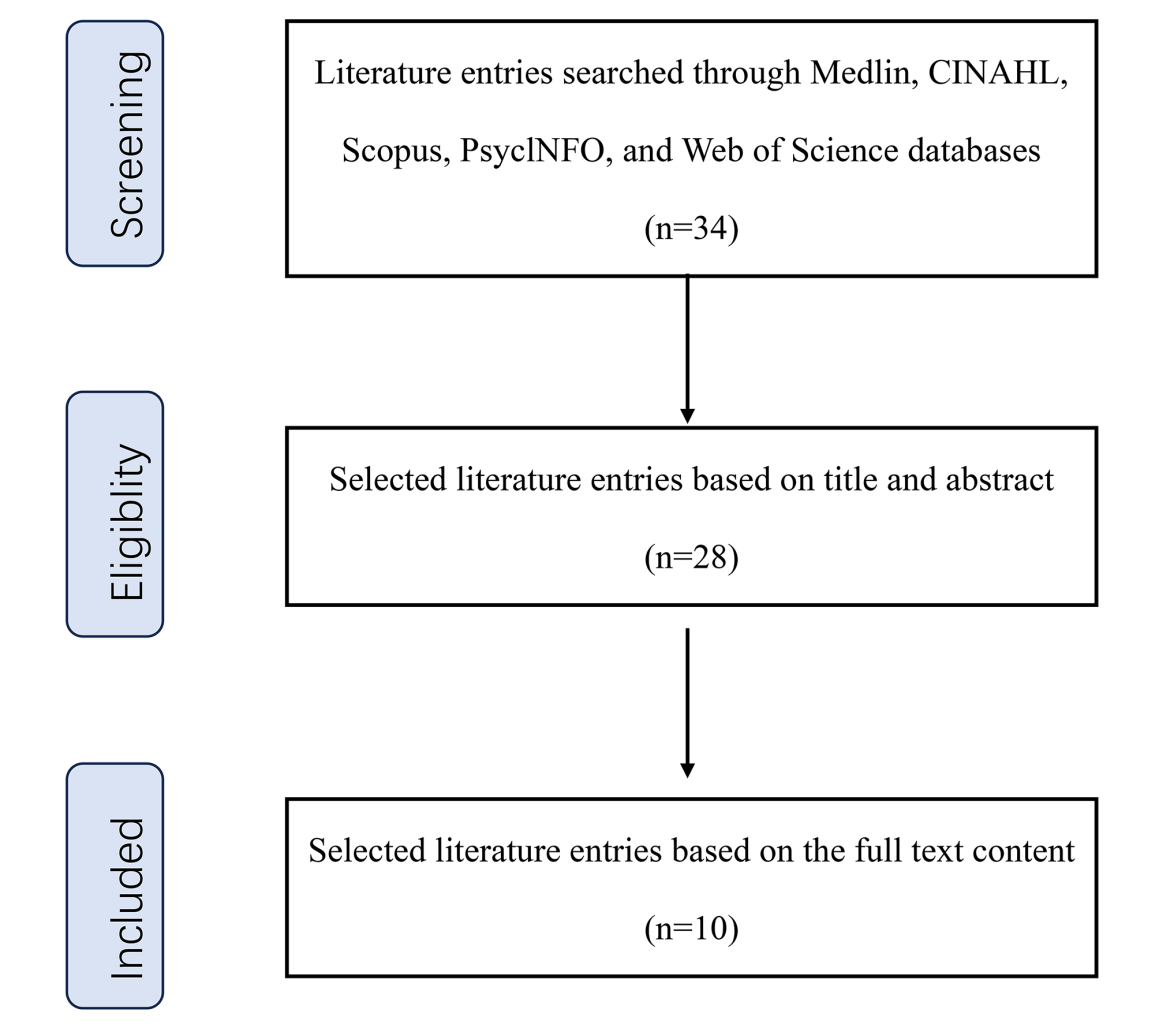
PRISMA protocol

We searched five electronic databases (Medline, CINAHL, Scopus, PsychINFO, and Web of Science) in January 2020 for publications from 2011 to 2020 using the following keywords: “entrustability and psychiatry” and “entrustable professional activit* and psychiatry,” retrieving 34 publications. We screened them for suitability based on their titles and abstracts, searching for articles on “confidence in professional behavior (EPA).” Twenty-eight papers remained after the first screening step. We assessed their suitability based on a full-text review, searching for publications with specific EPAs. Ten articles remained after this screening(Fig. 1). We limited the search to articles written in English, the type of literature is narrative.

The ten articles contained 61 EPAs, including duplicates. Following the 13 EPAs mentioned in a published guide from 2020 [19], we removed duplicates, summarized, and merged items, leading to the selection of 12 EPAs.

#### Preparing the expert inquiry form

Based on the literature review, referring to the 13 EPAs mentioned by the Association of American Medical Colleges Core Entrustable Professional Activities for Entering Residency, the 20 EPAs suggested by the Royal College of Physicians and Surgeons of Canada, and the rotation mode of standardized psychiatry training detailed in the Rules for Standardized Resident Training in Beijing (2013 version) [23], we developed a phase-based modular EPAs suitable for the three rotation stages, that is, the general, specialized, and mobile stages. The first draft expert consultation form for EPAs of psychiatry residents was formed after several discussion rounds within the research group and included 19 EPA indicators.

#### Selecting the consulting experts

We adopted an objective sampling method to select 44 experts from nine Grade-A psychiatry departments in Beijing, Tianjin, Shanxi, Heilongjiang, Henan, Zhejiang, Sichuan, and Yunnan Provinces. The expert inclusion criteria were: (i) bachelor’s degree or higher; (ii) a deputy senior professional title or higher; (iii) over ten years of clinical work and teaching experience; (iv) participated in the standardized training of psychiatry residents; and (v) able to complete at least two expert consultation rounds. All experts provided their informed consent for participation in this study.

#### Implementing the expert consultation

We distributed the consultation questionnaires as Questionnaire Star and conducted two consultation rounds, 2 to 3 weeks apart. In the first consultation round, The first part of the questionnaire is to introduce to the experts EPA concepts, the study background information, its purpose, and a preliminarily constructed evaluation index system and asked them to score the importance and familiarity levels of the indicators. We used a Likert 5-level scoring method, assigning scores from one point (very unimportant/unfamiliar) to five points (very important/familiar). The questionnaire also collected basic information on the experts, their degree of familiarity with the subject, and the basis for their judgment. We sorted, summarized, and statistically analyzed the returned expert opinions of the first round to generate the form for the second round of expert consultation, which was based on the results of the first round. We asked experts to evaluate the observability, authenticity, multiple competencies, effectiveness, repeatability, and feasibility of the indicators. Experts graded the entrustable level that should be achieved at the end of each stage and at the end of the course, referring to the five-level entrustable assessment scale [5] and Twelve tips to develop entrustable professional activities [20] proposed by ten Cate. Level 1 is “no permission to execute the EPA,” indicating that the resident’s knowledge and skills are not enough to implement the EPA and must be completed by the supervisor. Level 2 is “execute EPA under full supervision,” indicating that the resident could execute the EPA but required full supervision and guidance. Level 3 is “execute EPA under occasional guidance” indicating that the resident could execute the EPA, but the supervisor needs to respond to the resident’s needs, provide timely help, and review the outcomes. Level 4 is “execute EPA independently, without supervision,” indicating that the resident can work independently, knows the risks, and can perform safely without supervision. Level 5 is “guide others to execute EPA,” indicating that the resident can supervise others when implementing the EPA. We summarized and statistically analyzed the returned expert opinions of the second round. As the statistical analysis results showed consistent feedback results after the two expert consultation rounds, the consultation ended.

#### Index screening criteria

We calculated the mean and coefficient of variation (CV) of each index following a expert opinions. The index screening criteria were importance score > 3.75 and CV < 0.25. The research group revised the EPAs following the questionnaire results and expert opinions.

### Expert enthusiasm, authority, coordination, and concentration

The effective questionnaire recovery rate expressed the expert enthusiasm degree.

The authority coefficient, determined by the experts’ judgment basis and familiarity degree coefficients, expressed the expert authority degree. We used the following formula: authority coefficient = (familiarity degree coefficient + judgment basis coefficient) / 2 [24]. Values above 0.7 indicated good expert authority.

The CV and Kendall harmony coefficient expressed the degree of coordination among the expert opinions. The smaller the CV, the more consistent the expert opinions. The greater the Kendall harmony coefficient, the better the coordination among the expert opinions.

The arithmetic mean and the full score rate reflected the concentration of expert opinions.

### Statistical methods

For statistical analysis, we used IBM SPSS Statistics for Windows, Version 26.0 (IBM Corp., Armonk, NY, USA). The measurement data are expressed as means (standard deviations), and counting data are expressed as frequencies and percentages. The mean, full score ratio, and CV described the index scores. We considered differences with *P* < .01 to be statistically significant.

## Results

### Basic experts’ information

We consulted 44 experts. Their mean (standard deviation) age was 40.6 (5.3) years, and they had engaged in standardized resident training for 10.9 (4.6) years. The experts included three with a PhD degree (6.8%), 27 with a master’s degree (61.4%), and 14 with a bachelor’s degree (31.8%). Fifteen (34.1%) had full senior professional titles, and 29 (65.9%) had deputy senior professional titles. They included five residential training directors, nine teaching and research department directors, six teaching secretaries, five clinical department directors, eight ward directors, and 11 clinical teachers.

### Expert enthusiasm, authority, coordination, and concentration

The effective questionnaire recovery rate in the two consultation rounds was 100% (44/44), indicating that the experts paid attention and had a high degree of enthusiasm for this study.

The expert authority coefficients of the first and second consultation rounds were 0.861 and 0.881, respectively. The expert familiarity and judgment basis coefficients were 0.818 and 0.904 in the first round and 0.847 and 0.915 in the second round, respectively.

In the two rounds of consultation in this study, except for one index, the coefficient of variation of the evaluation results of each index was between 0.05 and 0.24. The Kendall harmony coefficients of the first and second expert consultation rounds were 0.279 (χ^2^ = 405.43, *P* < .001) and 0.389 (χ^2^ = 3456.83, *P* < .001), respectively, indicating that the coordination degree of expert opinions was good.

The arithmetic means of the various indicators’ evaluation results in the two consultation rounds ranged between 3.61 and 4.93, and the full score rates were between 13.6% and 93.2%.

### The establishment and validation of EPAs for psychiatric residents

Following the experts’ opinions, we deleted or modified some contents of the various EPAs, resulting in 62 content descriptions. After the first round of expert consultation, we deleted 15 content descriptions and modified ten. After the second round of expert consultation, we revised four content descriptions; the final content descriptions are presented in supplementary Table S1. See Table 1 for competencies [25] related to the 17 EPAs.

**Table 1:** Evaluation index of entrustable professional activities of psychiatric residents at stages

Cultivation stage	Rotation department	Duration (month)	EPA index	Relevant competencies^a[22]^
General stage	Neurology	4	Identification and treatment of common neurological diseases	PC2, PC4, KP1, KP2, KP3, KP4, ICS1, IC2, IC7, P1, P3, P5, PBLI1, PPD8
	Emergency	3	Identification and treatment of common emergency diseases	PC2, PC4, KP1, KP2, KP3, KP4, ICS1, IC2, IC7, P1, P3, P5, PBLI1, PPD8
	Cardiovascular	2	Identification and treatment of common cardiac diseases	PC2, PC4, KP1, KP2, KP3, KP4, ICS1, IC2, IC7, P1, P3, P5, PBLI1, PPD8
Specialized stage	Psychiatric intensive care unit	12	Clinical psychiatric communication	PC5, PC11, ICS1, ICS2, ICS3, ICS4, ICS6, ICS7, P1, P2, P3, P4, P5, P6, PPD7
			Writing medical psychiatric documents and case reports	PC4, PC6, ICS1, ICS2, ICS5, P4, SBP1
			Diagnosis and treatment of severe adult mental disorders	PC2, PC4, KP1, KP2, KP3, KP4, ICS1, IC2, IC7, P1, P3, P5, PBLI1, PPD8
			Identification and treatment of critical psychiatric illnesses	KP1, KP2, KP3, KP4, KP5, PPD2, PPD4, PPD8, SBP3, IPC3
			Selection and clinical use of psychotherapy	PC5, PC6, PC11, KP3, KP4, KP5, KP6
	Psychiatric light illness ward, clinical psychology department, or open ward	6	Diagnosis and treatment of mild adult mental disorders	PC2, PC4, KP1, KP2, KP3, KP4, ICS1, IC2, IC7, P1, P3, P5, PBLI1, PPD8
			Quantitative psychological assessment and judgment	PC1, PC2, PC4
			Using psychotherapy methods	PC5, PC10, KP5, P1, P3, P5, P6, SBP1
	Rehabilitation or geriatric psychiatry department or child and adolescent psychiatric ward	3	Diagnosis and treatment of senile mental disorders	PC2, PC4, KP1, KP2, KP3, KP4, ICS1, IC2, IC7, P1, P3, P5, PBLI1, PPD8
			Diagnosis and treatment of mental disorders of children and adolescents	PC2, PC4, KP1, KP2, KP3, KP4, ICS1, IC2, IC7, P1, P3, P5, PBLI1, PPD8
			Psychiatric rehabilitation guidance and health education	PC7, PC9, ICS1, ICS2, ICS3, ICS4, P4, SBP4, IPC2, KP4, PPD7
	Outpatient and emergency psychiatry	3	Evaluation, admission, and treatment of psychiatric outpatients	PC3, PC5, PC6, PC7, PC8, KP4, PBLI1, ICS6, P6, IPC1, IPC2, PPD7
Mobile stage	MECT treatment room or other rotation departments	3	MECT treatment	PC1, PC7, ICS5, ICS6, P6, SBP3, PPD7
			Guiding beginners	PBLI1, PBLI2, PBLI3, PBLI5, PBLI8
^a^Based on the research group’s consensus; the listed competencies’ abbreviations follow the general physician competency reference list.				

EPA, Entrustable professional activity; MECT, Modified electroconvulsive therapy

The importance, familiarity, observability, authenticity, multiple competencies, effectiveness, repeatability, and feasibility values were all greater than 3.5. Except for the observability of “guiding beginners,” the indicators’ CV values were all smaller than 0.25, indicating that the indicators’ setting was reasonable (Table 2).

**Table 2:** Results of the expert consultation on the entrustable professional activity indexes of psychiatry residents

EPA	Importance		Familiarity		Observability		Authenticity		Multiple competencies		Effectiveness		Repeatability		Feasibility	
	Mean	CV	Mean	CV	Mean	CV	Mean	CV	Mean	CV	Mean	CV	Mean	CV	Mean	CV
1. Identification and treatment of common neurological diseases	4.45	0.11	3.86	0.21	3.61	0.24	3.84	0.23	3.86	0.22	3.98	0.19	3.98	0.16	3.95	0.19
2. Identification and treatment of common emergency diseases	4.59	0.14	3.95	0.18	3.64	0.24	3.84	0.24	4.02	0.18	4.07	0.17	3.80	0.21	3.95	0.18
3. Identification and treatment of common cardiac diseases	4.43	0.16	3.70	0.21	3.70	0.22	3.93	0.19	3.93	0.16	4.05	0.19	3.98	0.17	3.98	0.19
4. Clinical psychiatry communication	4.91	0.06	4.68	0.11	4.09	0.21	4.23	0.18	4.25	0.18	4.25	0.17	4.20	0.18	4.32	0.17
5. Writing medical psychiatry documents and case reports	4.82	0.08	4.66	0.11	4.11	0.24	4.27	0.19	4.32	0.16	4.23	0.18	4.25	0.16	4.34	0.16
6. Diagnosis and treatment of severe adult mental disorders	4.86	0.07	4.68	0.11	4.00	0.22	4.30	0.17	4.25	0.17	4.25	0.19	4.20	0.17	4.18	0.17
7. Identification and treatment of critical psychiatric illnesses	4.93	0.05	4.61	0.13	3.89	0.24	4.07	0.20	4.14	0.19	4.23	0.18	4.02	0.19	4.07	0.19
8. Selection and clinical use of psychotherapy	4.77	0.09	4.55	0.14	4.07	0.23	4.08	0.18	4.27	0.17	4.20	0.18	4.23	0.17	4.23	0.19
9. Diagnosis and treatment of mild adult mental disorders	4.57	0.13	4.45	0.15	4.00	0.22	4.09	0.19	4.11	0.19	4.20	0.18	4.14	0.17	4.09	0.18
10. Quantitative psychological assessment and judgment	4.09	0.17	3.91	0.21	3.86	0.22	4.07	0.18	3.95	0.20	4.05	0.17	4.07	0.20	4.11	0.15
11. Using psychotherapy methods	4.07	0.14	3.75	0.21	3.68	0.24	3.86	0.23	3.80	0.20	3.95	0.20	3.93	0.20	3.89	0.19
12. Diagnosis and treatment of senile mental disorders	4.57	0.12	4.07	0.19	3.95	0.24	4.16	0.20	4.07	0.21	4.11	0.19	4.14	0.16	4.05	0.21
13. Diagnosis and treatment of mental disorders of children and adolescents	4.55	0.12	4.05	0.18	3.98	0.23	4.18	0.18	4.09	0.20	4.05	0.23	4.11	0.19	4.09	0.19
14. Psychiatric rehabilitation guidance and health education	4.27	0.15	4.02	0.17	3.84	0.23	3.95	0.18	4.09	0.18	4.11	0.16	4.11	0.18	4.05	0.18
15. Evaluation, admission, and treatment of psychiatric outpatients	4.52	0.13	4.36	0.16	3.86	0.22	4.07	0.18	4.11	0.19	4.20	0.16	4.05	0.20	4.07	0.17
16. MECT treatment	4.32	0.18	4.16	0.21	3.82	0.24	4.05	0.21	4.09	0.20	4.11	0.19	4.18	0.18	4.14	0.17
17. Guiding beginners	4.20	0.23	4.20	0.20	3.68	0.28	3.93	0.21	4.02	0.18	3.89	0.23	3.91	0.21	4.00	0.20

EPA, entrustable professional activity; CV, coefficient of variation; MECT, Modified electroconvulsive therapy

### Expected levels of supervision on the EPA for psychiatry residents at their various training stages

After the two consultation rounds, the expected levels of supervision on the EPAs at the various stages were similar. The expected levels of supervision CVs of the EPA indicators at the end of each stage and at graduation were all under 0.25 (Table 3).

**Table 3:** Expected entrustable levels of entrustable professional activities of psychiatry residents at various stages

EPA	End of general stage		End of pecialized stage		End of mobile stage		At graduation	
	Mean	CV	Mean	CV	Mean	CV	Mean	CV
1. Identification and treatment of common neurological diseases	3.27	0.22					4.11	0.16
2. Identification and treatment of common emergency diseases	3.11	0.22					3.91	0.17
3. Identification and treatment of common cardiac diseases	3.18	0.23					3.91	0.19
4. Clinical psychiatric communication			3.57	0.18			4.45	0.14
5. Writing medical psychiatric documents and case reports			3.66	0.18			4.59	0.12
6. Diagnosis and treatment of severe adult mental disorders			3.45	0.19			4.41	0.14
7. Identification and treatment of critical psychiatric illnesses			3.23	0.19			4.02	0.16
8. Selection and clinical use of psychotherapy			3.32	0.20			4.20	0.16
9. Diagnosis and treatment of mild adult mental disorder			3.52	0.17			4.41	0.13
10. Quantitative psychological assessment and judgment			3.41	0.20			4.20	0.17
11.Using psychotherapy methods			3.11	0.22			3.86	0.19
12.Diagnosis and treatment of senile mental disorders			3.25	0.23			3.98	0.16
13.Diagnosis and treatment of mental disorders of children and adolescents			3.30	0.23			3.91	0.18
14.Psychiatric rehabilitation guidance and health education			3.64	0.16			4.52	0.13
15.Evaluation, admission and treatment of psychiatric outpatients			3.25	0.23			4.07	0.17
16.MECT treatment					3.11	0.24	4.00	0.21
17.Guiding beginners					3.30	0.21	4.14	0.16

EPA, entrustable professional activity; CV, coefficient of variation; MECT, Modified electroconvulsive therapy

## Discussion

### The EPA index for psychiatry residents is scientifically valid

In methodology terms, we adopted a literature review and the Delphi expert consultation method to formulate and select indicators following scientific standards. We invited experts from nine psychiatric hospitals across the country that included experts in teaching management, teaching research, and clinical teaching. Most experts had a graduate degree, senior title, and rich experience in resident training, teaching, and management. The experts consulted in this study showed good authority and enthusiasm. The CV and expert coordination coefficient of all EPA indicators were good, making the research results scientifically valid.

### Phase-based modular characteristics of the EPAs in the psychiatry department

Relatively few studies on EPAs have been conducted in China; exceptions include, however, the research on constructing resident EPAs by Qi et al. of Peking University Hospital [26] and an obstetrics and gynecology EPA study [27]. The former flatly disassembled clinical behaviors into 17 key behaviors, while the latter listed key technical operations in obstetrics and gynecology as EPA indicators. However, the above-mentioned local EPA evaluation systems could not meet the particular training objective requirements at various stages and the standardized psychiatry training rules.

We adopted phase-based modular thinking to match the standardized training characteristics in the psychiatry department when constructing the EPA indicators, making them suitable for the clinical environment. The phase-based modularization also makes them observable, repeatable, and feasible. The formulated EPAs are more suitable for the specific characteristics of psychiatry specialty training than the clinical resident EPAs, and could be used when exploring EPAs for other specialty training programs. Unlike the standardized training of clinical residents, the psychiatry department EPAs were constructed into five modules, including clinical psychiatry communication, quantitative psychological assessment and judgment, using psychotherapy methods, evaluation, admission, and treatment of psychiatry outpatients, and modified electroconvulsive therapy treatment, highlighting the characteristics of the specialty. Psychiatrists serve patients with mental diseases, who face obstacles to understanding and affection. Establishing a treatment relationship with patients, reflecting their understanding, providing humanistic care, and communicating effectively are essential skills of psychiatric clinical work [28]. The diagnosis and treatment at the psychiatry department differ from those at other clinical medicine departments. They rely more on detailed medical history, professional mental examination and consultation, and necessary psychological examination scales. Psychiatry lacks the gold standard objective examination of internal medicine and surgery, making diagnosis and evaluation more varied and subjective [29]. Therefore, using psychological examination scales is particularly important and indispensable. Psychiatry residents should master selecting and interpreting common psychological scales. The particularity of psychiatry compared with other clinical disciplines is highlighted by its theoretical basis and clinical practice. The discipline’s nature originated from two equally important “genes,” natural and social sciences. Therefore, some scholars believe that psychiatrists should have certain psychological treatment abilities in addition to being able to perform biomedical diagnosis and treatment, so that they can learn to walk in both realms [30]. Another prominent feature of psychiatry is risks other than the disease itself, including behaviors that endanger society, other people, or the patient through self-inflicted injuries and suicidal behaviors. The mental health law defines the principles of involuntary hospitalization for six serious mental disease types, establishes a mental health monitoring network, and implements a reporting system for serious mental disorders [31]. Therefore, psychiatrists working at the psychiatry outpatient department should first assess and judge the risks of impulsive or self-inflicted injury and suicidal behaviors and treat these according to the mental health law provisions, in addition to undertaking routine diagnostic and therapeutic work. The EPAs under the above five modules obtained high scores in the expert consultation importance evaluation and had good consistency. Evidently, the EPAs constructed in this study are suitable for the overall objectives and discipline characteristics of standardized training of psychiatry residents, making them highly practical.

### The localized nature of the EPA indicators for the psychiatry department

Boyce et al. [10] developed four EPAs for first-year residents using a questionnaire survey, making it an exploratory study for psychiatry EPA construction. Young et al. [12] did not consider the impact of the clinical environment and disease category and severity on the EPA indicators when constructing their suggested 13 EPAs. However, they pointed out that these indicators were too broad. Weiss et al. [11] constructed EPAs by years, showing the phased and dynamic development of clinical EPAs.

The standardized training developed by Royal College of Canada is divided into four stages, including Transition to Discipline, Foundations, Core, and Transition to Practice [20], which is equivalent to the second stage of resident physician training in China, and the duration is also 2 years. Our research is currently focused on the standardized training phase for resident physicians, which is the rotation phase of the first three years. Therefore, there are still certain differences in the phased training at home and abroad.

The training of resident psychiatrists in Beijing is conducted through rotation between psychiatry and related departments. The rotation is divided into three stages: general department stage (9 months), specialized department stage (24 months), and mobile stage (3 months). The rotation of related departments includes Neurology, emergency department or intensive care unit, and cardiovascular medicine department. The rotation of psychiatry includes psychiatric intensive care unit, psychiatric mild illness ward or clinical psychological department or open ward, community, rehabilitation department or geriatric department, or pediatric department, and psychiatric outpatient and emergency department, The mobile phase includes clinical research or other rotating departments.

Therefore, simply dividing by years or establishing EPAs based solely on foreign phased training systems cannot meet the standardized training requirements for resident physicians in China.

Our research and the standardized psychiatry training characteristics led to the construction of EPAs in a phase-based and modular manner, explaining the final 17 EPA indicators. Therefore, the proposed EPA set is suitable for the practice and clinical environment of standardized psychiatry training in China and has good observability and measurability.

### The limitations of our research

In this study, we selected experts from nine psychiatric hospitals in various regions of China. Based on the detailed psychiatry training rules in Beijing, this study represents the current mode and characteristics of standardized psychiatry training. However, the consultation scope should be expanded further with a view to building and implementing an EPA evaluation system for psychiatry departments nationwide.

Our research has certain limitations. Firstly, due to participating in the Delphi expert consultation method, all mentors in the field of psychiatry were selected, and the subjects of consultation did not cover resident physicians, lacking a perspective as a guide, which may lead to a certain bias in the formulation of EPAs. Secondly, due to factors such as time and the impact of the epidemic, it was not possible to invite international experts to participate in this study. Thirdly, international standard for evaluating EPAs were not used.

### Future research directions on psychiatry EPAs

We propose these future research directions: (i) compare the EPA and existing evaluation systems to further optimize the EPA evaluation system; (ii) focus on implementing and applying the EPAs in standardized psychiatry training; (iii) training courses should be developed following the evaluation indicators and the training-stage objectives, evaluating the indicators in evaluator and resident groups; and (iv) for educators, analyze and compare the EPA evaluation system with the existing Objective Structured Clinical Examination structured assessment system and analyze the relevant factors to provide some reference for further exploration of the training approach to combine standardization, homogeneity, and individualization.

## Conclusion

We used a literature review and the Delphi method to establish a phase-based modular EPA index for psychiatry residents at their various training stages. These psychiatry department EPAs provide a simple and feasible competency evaluation method for resident training and clarify the training objectives for residents, clinical teachers, and management personnel. We intend to continue exploring quantitative research on the effectiveness of the psychiatry EPA evaluation system, with a view to establishing a standardized scheme for their implementation and evaluation.

### Electronic supplementary material

Below is the link to the electronic supplementary material.

Supplementary Material 1

## Data Availability

The datasets generated and/or analyzed during the current study are available from the corresponding author on reasonable request.

## Declarations

### Authors’ information

FY is the director of the Department of Psychosomatic Medicine of Beijing Huilongguan Hospital, XY is the director of the quality control office of the medical department of Beijing Huilongguan Hospital, LZ is the director of the education department of Beijing Huilongguan Hospital, HC is the deputy director of the National Medical Education Development Center of Peking University, LB is the deputy director of the medical office of Beijing Huilongguan Hospital, and FY is the secretary of the Party Committee of Beijing Huilongguan Hospital.

## Abbreviations

CBME: Competency-based medical education
CV: Coefficient of variation
EPAS: Entrustable professional activities

## References

[1] Frenk J, Chen L, Bhutta ZA (2010). Health professionals for a new century: transforming education to strengthen health systems in an interdependent world. Lancet.

[2] Zhe J, Xin Q, Haichao L (2019). Application of entrustable professional activities in clinical medical education. Chin J Med Educ.

[3] Rekman J, Gofton W, Dudek N, Gofton T, Hamstra SJ (2016). Entrustability scales: outlining their usefulness for competency-based clinical assessment. Acad Med.

[4] Sterkenburg A, Barach P, Kalkman C, Gielen M, ten Cate O (2010). When do supervising physicians decide to entrust residents with unsupervised tasks?. Acad Med.

[5] Ten Cate O (2005). Entrustability of professional activities and competency-based training. Med Educ.

[6] Chen HC, van den Broek WE, ten Cate O (2015). The case for use of entrustable professional activities in undergraduate medical education. Acad Med.

[7] Xinhang C, Hongbin W, Jiang Z (2020). The development of entrustable professional activities research in medical education and its implications. Chin J Med Educ.

[8] Englander R, Carraccio C (2014). From theory to practice: making entrustable professional activities come to life in the context of milestones. Acad Med.

[9] Pinilla S, Lenouvel E, Strik W et al. Entrustable Professional Activities in Psychiatry: a systematic review. Acad Psychiatry 2020:44: 37–45. 10.1007/s40596-019-01142-7. 10.1007/s40596-019-01142-7 31732885

[10] Boyce P, Spratt C, Davies M, McEvoy P (2011). Using entrustable professional activities to guide curriculum development in psychiatry training. BMC Med Educ.

[11] Weiss A, Ozdoba A, Carroll V, DeJesus F (2016). Entrustable professional activities: enhancing meaningful use of evaluations and milestones in a psychiatry residency program. Acad Psychiatry.

[12] Young JQ, Hasser C, Hung EK (2018). Developing end-of-training entrustable professional activities for psychiatry: results and methodological lessons. Acad Med.

[13] Griffeth BT, Brooks WB, Foster A (2017). A psychiatric-specific entrustable professional activity for the evaluation of prospective psychiatric residents: towards a national standard. MedEdPORTAL.

[14] Klapheke M, Johnson T, Cubero M. Assessing entrustable professional activities during the psychiatry clerkship. Acad Psychiatry. 2017;41:345–349. 10.1007/s40596-017-0665-9. Epub 2017 Mar 17. PMID: 28315194. 10.1007/s40596-017-0665-9 28315194

[15] Large MM, Ryan CJ (2014). Suicide risk categorisation of psychiatric inpatients: what it might mean and why it is of no use. Australas Psychiatry.

[16] Martin A, Jacobs A, Krause R, Amsalem D (2020). The mental status exam: an online teaching exercise using video-based depictions by simulated patients. MedEdPORTAL.

[17] Port N, Weiss A, Maudsley I. Electroconvulsive therapy training: Can it be a model of an entrustable professional activity in a competency program? Australas Psychiatry. 2012;20:242–245. 10.1177/1039856212447879. Epub 2012 May 30. PMID: 22652309. 10.1177/1039856212447879 22652309

[18] Spratt C, Boyce P, Davies M, Bhugra D, Malik A (2011). The australian and New Zealand experience. Workplace-based assessments in Psychiatric Training.

[19] Hung EK, Jibson M, Sadhu J (2021). Wresting with implementation: a step-by-step guide to implementing entrustable professional activities (EPAs) in psychiatry residency programs. Acad Psychiatry.

[20] The Royal College of Physicians and Surgeons of Canada. Entrustable professional activities for psychiatry. https://www.royalcollege.ca/rcsite/home-e. Accessed January 16, 2019.

[21] Hennus MP, Jarrett JB, Taylor DR, Ten Cate O. Twelve tips to develop entrustable professional activities. Med Teach. 2023;45(7):701–707. 10.1080/0142159X.2023.2197137. Epub 2023 Apr 7. PMID: 37027517. 10.1080/0142159X.2023.2197137 37027517

[22] Hasson F, Keeney S, McKenna H (2000). Research guidelines for the Delphi survey technique. J Adv Nurs.

[23] Beijing Health Bureau. Detailed Rules for Standardized Resident Training in Beijing, 2013. Peking Union Medical College Press. ; 2013. http://www.pumcp.com/portal/sites/xiehe/pages/index/index.html.

[24] Jinglou Q, Yaxin Z, Bo Q (2019). The Delphi method and its application in medical education research. Chin J Med Educ.

[25] Englander R, Cameron T, Ballard AJ (2013). Toward a common taxonomy of competency domains for the health professions and competencies for physicians. Acad Med.

[26] Xin Q, Zhe J, Xiaoning H (2021). Establishment of the entrustable professional activities for the residents. Chin J Med Educ.

[27] Tao L, Jun L, Ying L (2022). Establishment of standardized training and assessment model for residents in obstetrics and gynecology based on entrustable professional activities. Chin J Med Educ.

[28] Hongyu T, Yiru F, Psychiatry. Beijing: People’s Health Publishing House; 2014.

[29] Lin L (2018). Shen Yu Cun Psychiatry.

[30] Hongyu T (2015). We need psychiatrists who can see doctors. Chin Ment Health J.

[31] Yiming C (2013). Interpretation of mental health law. J Psychiatry.

