# Supplementary material for: Establishing entrustable professional activities for psychiatry residents in China
Source: BMC Med Educ. 2023 Sep 1;23:623. doi: 10.1186/s12909-023-04583-9 (PMC10474625; doi:10.1186/s12909-023-04583-9)
Supplement: Supplementary file 1 — Supplementary Material 1 [file 12909_2023_4583_MOESM1_ESM.docx]

**Table S1. Content description of the EPAs for psychiatry residents**

| **EPAs title** | **Content description** |
| --- | --- |
| 1. Identification and treatment of common neurological diseases | Physical examination of the nervous system |
|  | Basic interpretation of head CT, MRI, and EEG report |
|  | Identification and diagnosis of common neurologic diseases (cerebrovascular, central nervous system infections, and cerebral degenerative diseases) |
|  | Simple treatment of common neurological diseases (cerebrovascular, central nervous system infections, and cerebral degenerative diseases) |
| 2. Identification and treatment of common emergency diseases | Clinical emergency cardiopulmonary resuscitation |
|  | Identification and simple treatment of common emergency situations |
|  | Use of common rescue drugs |
| 3. Identification and treatment of common cardiac diseases | ECG examination – operation and result interpretation |
|  | Interpretation of cardiac imaging |
|  | Interpretation of Holter monitor results |
|  | Diagnosis and basic treatment of common heart diseases, including hypertension, coronary heart disease, and arrhythmia |
|  | Rational use of electric defibrillation |
| 4. Clinical psychiatry communication | Answer family members' questions about diagnosis and treatment |
|  | Answer patients' questions about diagnosis and treatment |
|  | Discuss treatment plan with family members and patients, and establish treatment alliance |
|  | Communicate and inform the family members of changes in the patient’s condition or special conditions during treatment |
|  | Inform family members before special treatments |
| 5. Writing medical psychiatry documents and case reports | Comply with the requirements and specifications of medical records and record the medical records in a timely, accurate, and clear manner |
|  | Perform oral and written case reports as needed |
| 6. Diagnosis and treatment of severe adult mental disorders | Collect medical history independently and accurately |
|  | Correct mental examination |
|  | Accurately identify mental symptoms |
|  | Make a reasonable diagnosis and differential diagnosis |
|  | Assess corresponding risks |
|  | Give appropriate treatment plan |
|  | Adjust the treatment according to disease changes |
|  | Timely and accurately report according to the management of severe mental disorders |
| 7. Identification and treatment of critical psychiatric illnesses | Rapid and accurate identification of critical psychiatric patients |
|  | Preliminarily judge and analyze the potential causes of the patient's critical illness |
|  | Initiate initial treatment of critical patients in the psychiatry department |
|  | Timely report to the superior physician for help |
| 8. Selection and clinical use of psychotherapy | Rational selection of common antipsychotics, and identification and treatment of common side effects |
|  | Rational selection of commonly used antidepressants, and identification and treatment of common side effects |
|  | Rational selection of common emotion stabilizing drugs, and identification and treatment of common side effects |
| 9. Diagnosis and treatment of mild adult mental disorders | Collect medical history independently and accurately |
|  | Correct mental examination |
|  | Accurately identify mental symptoms |
|  | Make a reasonable diagnosis and differential diagnosis |
|  | Give an appropriate treatment plan |
|  | Rational selection of commonly used anti-anxiety drugs, and identification and treatment of common side effects |
|  | Rational selection of commonly used hypnotic drugs, and identification and treatment of common side effects |
| 10. Quantitative psychological assessment and judgment | Select the appropriate psychological test according to the patient's disease performance |
|  | A simple and correct interpretation of psychological test results |
| 11. Using psychotherapy methods | Select appropriate psychotherapy techniques for various diseases |
|  | Carry out at least one commonly used psychotherapy method (such as cognitive therapy, psychodynamics, supportive psychotherapy) |
|  | Write records of psychotherapy cases |
| 12. Diagnosis and treatment of senile mental disorders | Diagnosis and treatment of mental disorders caused by common organic diseases, including cerebrovascular and somatic diseases |
|  | Diagnosis, differentiation, and treatment of common dementia (AD and VD) |
| 13. Diagnosis and treatment of mental disorders of children and adolescents | Identification and diagnosis of common mental disorders in children and adolescents |
|  | Drug selection and/or psychotherapy for mental disorders in children and adolescents |
| 14. Psychiatric rehabilitation guidance and health education | Instruct patients to conduct at least one kind of rehabilitation training, such as drug self-management and social function rehabilitation |
|  | Carry out corresponding health education according to various needs |
| 15. Evaluation, admission, and treatment of psychiatric outpatients | Conduct a risk assessment on outpatients (e.g., impulsive, suicidal, and self-injury risks), being aware of prevention methods and able to handle risks |
|  | Staying alert to possible public events related to mental illness and timely identifying them |
|  | Carry out relevant clinical treatments in compliance with the mental health law (e.g., involuntary hospitalization and medical protective constraint) |
|  | Timely and accurately reporting according to established procedures and requirements |
| 16. MECT treatment | Select MECT treatment according to the clinical needs |
|  | Participate in an MECT operation |
| 17. Guiding beginners | Carry out teaching activities in combination with clinical practice |
|  | Apply appropriate teaching methods |
|  | Make active and effective use of teaching resources |
|  | Be good at observing and providing feedback and guidance |

EPA, entrustable professional activity; CT, computed tomography, MRI, magnetic resonance imaging, EEG, electroencephalography; ECG, echocardiography; MECT, Modified electroconvulsive therapy; AD, Alzheimer disease; VD,Vascular dementia.
